# How fast did newborns die in Nigeria from 2009-2013: a time-to-death analysis using Verbal /Social Autopsy data

**DOI:** 10.7189/jogh.09.020501

**Published:** 2019-12

**Authors:** Alain K Koffi, Jamie Perin, Henry D Kalter, Joseph Monehin, Adeyinka Adewemimo, Robert E Black

**Affiliations:** 1 Institute for International Programs, Johns Hopkins Bloomberg School of Public Health, Baltimore, Maryland, USA; 2 Center for Child and Community Health Research, Department of Pediatrics, Johns Hopkins School of Medicine, Baltimore, Maryland, USA; 3 Office of Population, Health, Nutrition and Education, USAID Dhaka/ Bangladesh; 4 Department of Health Planning, Research, and Statistics, Federal Ministry of Health, Abuja, Nigeria

## Abstract

**Background:**

The slow decline in neonatal mortality as compared to post-neonatal mortality in Nigeria calls for attention and efforts to reverse this trend. This paper examines how socioeconomic, cultural, behavioral, and contextual factors interact to influence survival time among deceased newborns in Nigeria.

**Methods:**

Using the neonatal deaths data from the 2014 Nigeria Verbal/ Social Autopsy survey, we examined the temporal distribution of overall and cause-specific mortality of a sample of 723 neonatal deaths. We fitted an extended Cox regression model that also allowed a time-dependent set of risk factors on time-to-neonatal death from all causes, and then separately, from birth injury/birth asphyxia (BIBA) and neonatal infections, while adjusting for possible confounding variables.

**Results:**

Approximately 26% of all neonatal deaths occurred during the first day, 52.8% during the first three days, and 73.9% during the first week of life. Almost all deaths (94.4%) due to BIBA and about 64% from neonatal infections occurred in the first week of life. The expected all-cause mortality hazard was 6.23 times higher on any particular illness day for the deceased newborns who had a severe illness at onset compared to those who did not. While the all-cause mortality hazard ratio of poor vs wealthier households was 0.77 (95% confidence interval (CI) = 0.648-0.922), the BIBA mortality hazard ratio of households with no electricity was 1.79 times higher compared to households with electricity (95% CI = 1.180-2.715).

**Conclusions:**

The findings suggest the need for continued improvement of the coverage and quality of maternal and neonatal health interventions at birth and in the immediate postnatal period. They may also require confirmation in real-world cohorts with detailed, time-varying information on neonatal mortality.

Of the 5.4 million under-five child deaths globally in 2017, about 2.5 million occurred during the neonatal period, representing 47% of under-five deaths [1]. About 99% of neonatal deaths occur in low- and middle-income countries [2,3]. Many of the deaths in these countries happen at home and do not receive a birth or death certificate, and thus have never been registered, reported or investigated by the health system. As a result, countries often do not know the true numbers of deaths or the causes of these deaths and thus are unable to take the right actions to prevent other babies and mothers from dying [3].

Nigeria is among the countries that failed to achieve Millennium Development Goal 4, showing slow rate of decline in the under-five mortality rate over the past 15 years. Neonatal mortality rate in Nigeria was estimated at 33 deaths per 1000 live births in 2017 [1], accounting for 33% of deaths in children younger than five years, with the most frequent causes of death being preterm delivery complications, intrapartum related events, sepsis, and pneumonia [4,5]. As the third Sustainable Development Goal (SDG 3) calls for all countries to reduce neonatal mortality to at least as low as 12 deaths per 1000 live births, it is estimated that up to two thirds of newborn deaths can be prevented in countries such as Nigeria if known and effective health measures are provided at birth and during the first week of life [6]. To that end, knowledge of the biological causes of neonatal deaths and the circumstances in which the deaths occurred are important. Information on the exact timing of neonatal deaths, particularly by the distribution of the cause-specific deaths, remains critical to illuminating our understanding of the risk of deaths at birth and in the immediate postnatal period, and will help policy makers, program managers and health care providers prioritize urgently-needed interventions [3].

Numerous studies have reported the causes of neonatal deaths in developed and developing countries in the last few years [6,7]. A few studies have explored and reported on the timing of neonatal deaths in developing country settings. For instance, about a decade ago, Lawn et al. [4] reported the daily risk of death in the first month of life based on the analysis of 47 data sets from the Demographic and Health Surveys of different countries pertaining to the period between 1995 and 2003.The study faced the inherent limitations of data from household surveys, and the lack of information on the timing of cause-specific neonatal deaths. A more recent paper used a systematic review technique to provide the distribution of overall and cause-specific deaths in the first 28 days of life [8] but failed to present the circumstances in which these deaths occurred.

In the context of Nigeria, a general dearth of community-based studies on neonatal mortality significantly limits our understanding of the breadth and depth of the problem for evidence-based programming. In addition, there has been a growing demand for mortality data to be disaggregated by gender, geographic location and socioeconomic status, to enable programs not only to improve resource allocation, but also for monitoring and evaluation purposes [8].

The current paper envisions to fill some of these gaps and aims to shed light on the timing of overall and cause-specific neonatal deaths in Nigeria, as well as on how socioeconomic, cultural, behavioral, and contextual factors interacted to influence survival time among deceased newborns. In other words, in the absence of a control survivor group, this study aims to investigate factors associated with immediate and early death among deceased newborns in Nigeria in 2009-2013.

## METHODS

### Study sites and sample

Verbal/social autopsy (VASA) interviews were conducted as a follow-up study on newborn deaths identified by the 2013 Nigerian Demographic and Health Survey (NDHS) of 38522 households undertaken by the National Population Commission from February to June 2013 [9]. The VASA study aimed for a sample of 986 neonatal deaths (0-27 days old) and 2268 child deaths aged 1-59 months (total 3254 deaths). This was based on precision of ±0.05 around the point estimate for the most common cause of young child deaths and ±0.07 for neonatal deaths, based on an assumed proportion of 50%, and assumed 10% loss due to household relocation and refusals to participate.

The NDHS identified all deaths of children in the prior 10 years from a full birth history of all women age 15-49 years. To limit issues related to faulty recall, while obtaining an adequate sample size, the VASA study examined deaths of children up to 59 months of age within the most recent 5 years, during which there were 1206 neonatal (0-27 days) deaths and 2779 child (1-59 months old) deaths. However, to minimize the interview burden on households and because of the likely overlap of social and health system determinants of death within households, the VASA study selected for interview one neonatal or child death at random within each household that reported one or more child deaths. This resulted in 3254 households being sampled, 2944 (90.5%) of which had a completed VASA interview of which 164 were determined to be stillbirths, 723 were neonatal deaths and 2057 were child deaths. The current analysis includes the 723 neonatal deaths only.

### Data collection tools and VASA interview

The description of the data collections tools, the training of trainers and interviewers and the conduct of the VASA interview have been provided in prior papers [6,10]. In summary, the VASA questionnaire blends the Population Health Metrics Research Consortium (PHMRC) verbal autopsy questionnaire to determine the biomedical cause of death, with the Child Health Epidemiology Reference Group (CHERG) Pathway Analysis social autopsy (SA) questionnaire [11] to inquire about well-child and illness events leading up to a death. The CHERG SA questionnaire updates an earlier Pathway Analysis instrument [12], in order to more fully examine the household, community and health system determinants of neonatal and child mortality, including maternal preventive and curative care in the event of a neonatal death or stillbirth.

The questionnaire was translated into Nigeria’s three major languages of Yoruba, Igbo, and Hausa which, in addition to English, are spoken by most persons in the study area. The translations were inserted into a Census and Survey Processing System (CSPro) software application (US Census Bureau, Suitland, USA) that was developed to enable direct, field-based CAPI (Computer Aided Personal Interview) capture of the VASA interview data on a netbook computer.

Data quality was not only ensured through the technical team trainers that also functioned as the quality controllers but was also monitored through field check tables generated by a CSPro program on the supervisors’ netbooks concurrently with fieldwork. This was an advantage since the technical team trainers (or field coordinators) could advise field teams of problems detected during fieldwork. Particularly, tables were generated to check various data quality parameters. The technical team trainers met in Abuja at the end of each monitoring visit to discuss the issues in the fieldwork and travelled to the states where immediate attention was required. Representatives from USAID and the National Population Commission also monitored fieldwork.

### Study outcome variable

In the current study, all the neonates died. The outcome of interest was the survival time or time to death, expressed in days for each newborn. We attempted to correct for the labeling of deaths with duration 0 days. It might happen that a neonate was born, got fatally sick and died on the first day of life. In such a case, the starting and ending days of his/her life would be equal, and the duration would be zero. Hence, to avoid this situation (zero for survival time), we added one day to the age at death of all deaths.

### Cause of deaths determination

The expert algorithm (EAVA) methods used by the VASA study for cause of death determination are described elsewhere [6]. In brief, the expert algorithms for the neonatal causes of death were based on those developed by verbal autopsy researchers for prior VA validation studies [6,13], further consultation and a literature review to identify illness signs and symptoms commonly associated with particular neonatal illnesses [14,15]. Hierarchies were developed for neonatal diagnoses to select the EAVA primary cause of death for each child from among all possible co-morbidities identified by the algorithms. To the extent possible, the ordering of the hierarchies was based on principles incorporated in the ICD-10. The algorithms and hierarchies utilized in this study, and their development, are described in a prior publication [16].

### Exploratory variables

The exploratory variables were grouped into individual (newborn), maternal, and household characteristics. The definition of each the variable is provided in **Table 1**.

**Table 1:** Definition and categorization of exploratory variables

Variable	Definition/categorization
**Household characteristics:**	
Household wealth	Household wealth index coded as poor = 1; rich = 0
Residence	Place of residence classified as rural = 1; urban = 0
Region of residence	Geopolitical zone of residence coded as north = 1; south = 0
Toilet facility	Recorded as unimproved = 1; improved (use of flush or improved pit toilet) = 0
Piped water	Household uses a piped or an improved water source, coded as no = 1; yes = 0
Electricity access	Access to electricity recorded as no = 1; yes = 0
**Maternal characteristics:**	
Maternal age	Maternal age (in years) at time of childbirth, coded as less than 30 years = 0; 30 years or more = 1 (Median maternal age at time of childbirth = 30 years)
Maternal education level	Highest educational level coded as no education = 1; Some primary or higher education = 0.
Parity	Number of children ever born, coded as Less than 4 children = 0; 4 children or more = 1 (Median number of children ever born in our sample = 4)
Quality antenatal care	The mother completed at least one quality ANC visit, that includes blood pressure checked, urine and blood sample taken, counseled about nutrition, and counseled about pregnancy danger signs; coded no = 0 and yes = 1
Place of delivery	Place where the mother delivered the baby coded as home or other = 0; health facility = 1
Any labor/delivery complication (Except preterm delivery complication)	The mother had intrapartum hemorrhage, probable maternal infection, eclampsia/preeclampsia, or prolonged labor, coded no = 0; yes = 1
**Child characteristics:**	
Gender:	Sex of the baby coded as boy = 1; girl = 0
Perceived illness severity at onset by syndrome	A scoring system based on respondents’ reports of the child’s feeding behavior, activity level and mental status. Coded as Normal/Mild/Moderate = 0; Severe = 1
**Main causes of death:**	
Birth Injury/ Birth Asphyxia (BIBA)	Baby died of BIBA. Coded as no = 0; yes = 1
Neonatal infections	Baby died of any neonatal infection, including sepsis, meningitis, pneumonia, tetanus, diarrhea. Coded as no = 0; yes = 1
Other causes	Baby died of other cause of death, including preterm birth complication or prematurity, congenital malformation, other or unspecified causes of death. Coded as no = 0; yes = 1

### Statistical analysis

In the descriptive analysis, we presented the hazard rate of neonatal death and the percentiles of survival times by individual, household and maternal characteristics among the deceased newborn, and the Wald chi2 test that tested the difference between categorical variables. The hazard rates are produced using the Kaplan-Meier approach in STATA 15.0 (StataCorp, College Station, TX, USA) in which the command *stsum* also provides the time at risk ***t*** along with the number (***n***) of individuals exposed. The hazard rate is thus ***n/t***.

The multivariate analyses were 2-fold: The first model, called the two-state model for survival data with one transient state “0: born alive” and one absorbing state “1: dead”, ie, a state from which further transitions cannot occur. In this study, all children were born (in state 0) and then died (state 1): that is, there is no censoring at all since every observation experienced the event of interest (‘death’). The objective of this model is to estimate the probability of having the event ‘death’ at time *t,* regardless of the cause of death, given that death has not occurred prior to that point.

In the second model, the “cause of death” ***h*** is observed in addition to the survival time ***t***. In this model, we dealt with one initial or transient state ‘Born alive: 0’, and **κ** mutually exclusive absorbing states, state ***h***, ***h = 1,…,κ*** corresponding to ‘death from cause ***h***’. The model is illustrated for ***κ = 3***, with ***κ = 1***, corresponding to birth injury/birth asphyxia (BIBA); ***κ = 2*** or neonatal infection that entails a more general non-specific categorization term including sepsis, meningitis, pneumonia, tetanus, diarrhea; ***κ = 3***, or all other causes of death, including preterm birth complication or prematurity, congenital malformation, other, or unspecified causes of death. But we did not include the ***κ = 3*** in the final model.

In both models, we first fit the Cox regression model to the data set before investigating the proportional hazards assumption. Of note, a feature of the Cox model is that the hazard or transition rates for different values of covariates are proportional, but the proportional hazard assumption needs to be tested [17,18]. An assessment of the proportional hazard’s assumption can be done by many numerical or graphical approaches [19,20]. None of these approaches are known to be better than the others in assessing whether the hazards are proportional or not. Because interpreting graphical plots can be arbitrary, and the conclusions highly dependent on the subjectivity of the researcher, we chose to use the numerical approach to assess the proportional hazards in this study to detect covariates that change over time [19]. Next, this test was accomplished by testing the correlation between the Schoenfeld residuals for a particular covariate and the ranking of the individual failure times [19]. The correlation near zero indicates that the proportional hazards assumption has not been violated (or the proportional hazard assumption has been upheld) [20]. For variables that violated the proportionality assumption, ie, so-called time-varying variables, we introduced interactions between those variables and ***t(log(t))*** that automatically corrected for the violation [17].

Of note, the 2013 Nigerian demographic and health survey data, which served as the platform to the VASA study, used a multistage, stratified sampling procedure with strata based on zonal (six geographic zones) and rural–urban divisions. Therefore, to ensure the sample was representative of the population, we used design-based women’s weights. We also used the Taylor linearization method for variance estimation. Statistical analyses were conducted with STATA survey commands *svy* in STATA 15.0 to adjust for the cluster sampling design, weights, and the calculation of standard errors.

## RESULTS

The basic characteristics of the deceased newborns are shown in **Table 2**. The total number of subjects is equal to 723, and the total analysis time at risk is 3977 days. The overall hazard rate here, is 723/3977 = 0.182. The percentiles of survival times were derived from the Kaplan-Meier survivor function. This function estimates about 25% neonatal death within 1 day, 50% within 3 days, and 75% within 8 days. The hazard rate of neonatal deaths in the southern regions was higher than those in the northern regions (0.216 vs 0.171, *P* = 0.011).

**Table 2:** Hazard rate of neonatal death and the percentiles of survival times by individual, household and maternal characteristics among the deceased newborn

	*n*		Time at risk (*t*) (in days)	Survival time percentiles			Hazard rate (*n*/*t*)	Wald χ^2^ (*P*-value)
25%	50%	75%
**All**	723		3977	1	3	8	0.182	
**Household characteristics::**								
South	212		980	1	3	7	0.216	6.46 (0.011)
North	511		2997	2	4	8	0.171	
**Household wealth:**								
Rich	348		1623	1	3	6	0.215	13.3 (0.0003)
Poor	375		2354	2	4	9	0.159	
**Residence:**								
Urban	202		1043	1	3	8	0.194	1.17(0.2789)
Rural	521		2934	2	3	8	0.178	
**Household has toilet::**								
Improved	154		791	1	3	7	0.194	0.64 (0.4249)
Un-improved	569		3187	2	3	8	0.179	
**Household used piped or improved water source:**								
Yes	234		1231	1	3	6	0.190	0.38(0.5395)
No	489		2746	1	4	8	0.178	
**Household had electricity:**								
Yes	316		1637	1	3	7	0.193	1.72 (0.1893)
No	407		2341	2	3	8	0.174	
**Maternal characteristics:**								
Maternal age (missing = 2):*								
<30 years old	354		1901	2	3	7	0.186	0.22 (0.6418)
30 years and more	366		2062	1	3	8	0.178	
**Education:**								
Some primary of higher education	342		1615	1	3	7	0.212	10.51(0.0012)
No education	381		2362	2	4	9	0.161	
**Parity: †**								
1-3 children	393		2250	2	3	8	0.175	1.00 (0.3167)
4 and more children	330		1727	1	3	7	0.191	
**Mother received antenatal visit of quality:**								
Yes	213		1100	1	3	8	0.193	0.73(0.3933)
No	510		2878	2	4	8	0.177	
**Place of delivery:**								
Institutional	263		1172	1	3	6	0.224	14.13 (0.0002)
Non-institutional	460		2805	2	4	9	0.164	
**Any labor/delivery complications:‡**								
No		494	2758	2	3	8	0.179	0.42 (0.5148)
Yes		229	1219	1	3	7	0.188	
Prolonged labor								
**Child characteristics:**								
Gender:								
Girl		312	1663	1	3	7	0.187	0.45 (0.5014)
Boy		411	2314	1	3	8	0.178	
**Perceived illness severity at onset by syndrome:**								
Moderate/Normal/ Mild		385	3054	3	6	11	0.126	86.61 (<0.0001)
Severe		323	899	1	2	3	0.360	
**Main causes of deaths:**								
BIBA		161	378	1	1	2	0.426	51.79 (<0.0001)
INFECTIONS§		410	2785	2	5	9	0.147	
OTHER CAUSES‖		152	815	2	3	7	0.187	

BIBI – birth injury/birth asphyxia

*Median Maternal age at time of childbirth = 30 years.

†Median number of children ever born in our sample = 4.

‡Except preterm delivery complication.

§Infections = Diarrhea, Meningitis, pneumonia, sepsis, tetanus.

‖Other Causes = congenital malformation, preterm birth/prematurity, other causes or unspecified.

A total of 348 neonatal deaths from rich households contributed to 1623 days of the analysis time at risk, whereas 375 neonatal deaths from poor households were in for 2354 days; thus, the hazard rate for richer households was significantly higher (0.215) compared to poorer households (0.159), *P* = 0.0003. Similarly, neonates born to mother with some primary or higher education, neonates born in health facilities, or neonates who had severe illness at onset had significantly higher hazard rates than their counterparts.

Among the study sample, 56.7% (or 410 out of 723) of deaths were attributable to infections, including diarrhea, meningitis, pneumonia, sepsis, and tetanus; 22.2% were from BIBA, while other causes (congenital malformation, preterm birth/prematurity, other causes or unspecified) represented 21.1% of the study sample.

**Figure 1:**
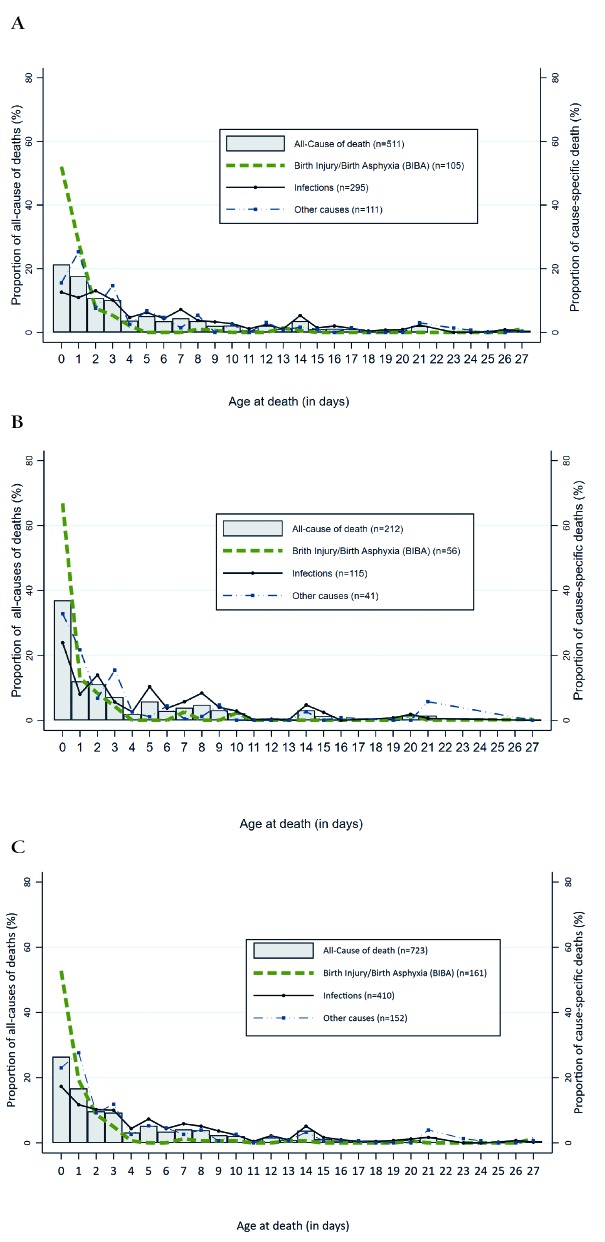
Distribution of neonatal deaths by age at death (0-27 days). Panel **A**. In northern regions of Nigeria 2009-13. Panel **B**. In southern regions of Nigeria 2009-13. Panel **C**. In Nigeria 2009-13.

**Figure 1** (panels A-c) depicts the distribution of overall and the three-main cause-specific (BIBA, infections and other causes) neonatal deaths by the “raw” age at death (0-27 days) variable. Approximately 26% of all neonatal deaths (37% in the South, vs 21.4% in the North) occurred during the first day and 73.9% during the first week of life (**Figure 1**, panel C). The first three days (day0-day2) of life alone accounted for more than one-half (52.8%) of the total neonatal deaths in the country. Almost all deaths (94.4%) due to BIBA occurred in the first week of life, with 93.1% of those deaths in the South and 95.2% in the North. The first day (day 0) alone contributed about two-thirds of the total BIBA deaths in the South and 51.9% in the North (**Figure 1**, panel B), making it to 80.4% and 80.3% by the second day of life in the South and North, respectively. About 64% of the total deaths secondary to infections occurred in the first week of life (68.1% in the South vs 62.2% in the North), and 25.9% of these deaths were claimed during the first two days with 32.0% in the South and 23.6 in the North).

**Figure 2:**
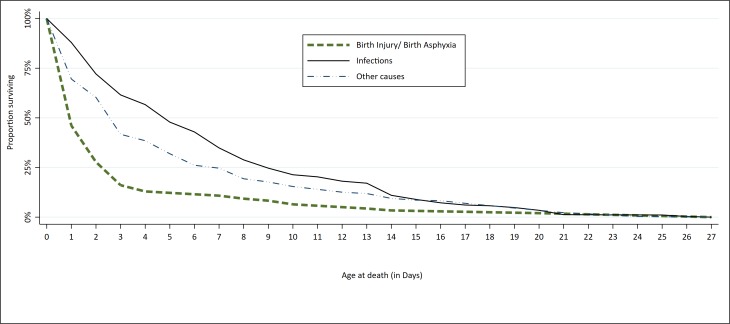
Kaplan-Meier survival estimates by age at death and cause-specific neonatal mortality, Nigeria 2009-13. *log-rank test for equality of survival curves: χ^2^ = 51.79; *P* < 0.001.

Overall, the hazard rates were 0.426, 0.1487 and 0.147 for BIBA, other causes and infections, respectively. The log-rank test revealed a statistically significant difference between the hazard rates of the three groups of causes of death (**Figure 2**).

The parameter estimates for neonatal death from all causes were generated in Cox regression procedure (prior to investigating the proportional hazards assumption) and are shown along with their *P*-values in **Table 3**. This table also exhibits the results of the test of proportional hazards assumption for each individual and global covariate. The correlation analysis of the partial residuals with time that the p- values obtained for some variables- toilet type in the household, whether the indexed child mother received any ANC or any ANC of quality, whether she delivered at a health facility, and whether the deceased child had a severe illness at onset were less than 0.05 (**Table 2**). In other words, these variables did not satisfy the proportional hazards assumption. For other variables used in the Cox regression model the proportional hazards assumption holds.

**Table 3:** Parameters estimates and test of proportional hazards assumption of neonatal death from all-causes in Nigeria

	Parameter estimates and *P* value		Test of proportional hazards assumption*			
	**Coefficient**	***P*-value**	**rho**	**χ-2**	**Degree of freedom**	***P*-value**
Household in the North	-0.022	0.828	0.044	1.49	1	0.222
Household in poor wealth group	-0.327	**0.001**	-0.059	2.20	1	0.138
Household in rural area	0.022	0.806	-0.059	0.71	1	0.400
Household had unimproved toilet	0.165	0.170	-0.032	6.50	1	**0.011**
Household used no piped or improved water source	-0.033	0.715	0.076	2.90	1	0.088
Household had no electricity	0.143	0.169	0.056	0.79	1	0.374
Mother’s age: 30 years or more†	0.020	0.840	-0.030	0.18	1	0.675
Mother had no education	-0.174	0.095	-0.014	0.30	1	0.587
Mother had high parity of 4 children or more‡	0.015	0.875	-0.018	0.60	1	0.438
Mother received no ANC or no quality ANC	0.101	0.366	-0.027	8.58	1	**0.003**
Mother did not deliver in a health facility	-0.084	0.369	0.080	5.75	1	**0.017**
Mother had any labor/delivery complications§	-0.031	0.730	-0.080	0.18	1	0.668
Deceased was a baby boy	0.007	0.928	-0.014	2.41	1	0.121
Deceased had a severe illness at onset	1.060	**0.000**	-0.053	23.71	1	**<0.001**
**Global test**				44.16	14	**0.0001**

*Robust variance-covariance matrix used in the test of proportional hazards assumption; The *estat phtest* [STATA 15.0 (StataCorp, College Station, TX, USA)] command tests, for individual covariates and as a whole, the null hypothesis of zero slope, which is equivalent to testing that the log hazard-ratio function is constant over time. Thus, rejection of the null hypothesis of a zero slope (see variables for which *P* value are in bold) indicates deviation from the proportional-hazards assumption.

†Median maternal age at time of childbirth = 30 y.

‡Median number of children ever born in our sample = 4.

§Except preterm delivery complication.

**Table 4** presents parameters estimates (along with *P*-value) of the neonatal deaths from all causes of deaths while considering the results of test of proportional hazards assumption. The first part of the equation reports the results for the time constant variables, and the second part of the equation contains the results for our time-varying covariates. After accounting for all of the variables, there were statistically significant associations between two variables-household wealth and severity of illness at onset- and all-cause mortality.

**Table 4:** Estimated hazards rates (with 95% confidence intervals) of neonatal death from all-causes of death, Nigeria

	All causes of death		
**Parameter estimate**	***P*-value‡**	**HR (95% CI for HR)**§
**Main co-variates**			
Household in the North	-0.056	0.573	0.95 (0.780-1.148)
Household in poor wealth group	-0.258	**0.004**	0.77 (0.648-0.922)
Household in rural area	0.056	0.523	1.06 (0.890-1.257)
Household had unimproved toilet	0.048	0.759	1.05 (0.772-1.426)
Household used no piped or improved water	-0.018	0.829	0.98 (0.836-1.155)
Household had no electricity	0.123	0.189	1.13 (0.941-1.359)
Mother’s age: 30 years or more	-0.012	0.899	0.99 (0.827-1.182)
Mother had no education	-0.135	0.167	0.87 (0.721-1.058)
Mother had high parity of 4 children or more	-0.011	0.908	0.99 (0.828-1.183)
Mother received no ANC or no quality ANC	-0.009	0.946	0.99 (0.773-1.271)
Mother did not deliver in a health facility	-0.088	0.481	0.92 (0.717-1.170)
Mother had any labor/delivery complications*	-0.007	0.927	0.99 (0.855-1.153)
Deceased was a baby boy	-0.002	0.982	1.00 (0.866-1.151)
Deceased had a severe illness at onset	1.830	**<0.001**	6.23 (4.522-8.589)
**Time varying co-variates (TVC):†**			
Household had unimproved toilet	0.001	0.992	1.00 (0.841-1.192)
Mother received no ANC or no quality ANC	0.082	0.298	1.09 (0.930-1.265)
Mother did not deliver in a health facility	-0.021	0.789	0.98 (0.843-1.138)
Deceased had a severe illness at onset	-0.697	**<0.001**	0.50 (0.406-0.611)

HR – hazards ratio, CI – confidence interval

*Except preterm delivery complication.

†Variables in TVC equation interacted with ***ln(_t)***; these are variables for which the null hypothesis of zero slope on the Test of proportional hazards assumption is rejected.

‡Significant *P* values (<0.05) are in bold.

§HR represents an increased (or decreased) risk of early neonatal death. HR>1.00 indicates increased risk of early neonatal death while HR<1.00 indicates reduced risk of early neonatal death; HR = 1 means the exposure/ characteristic/factor has no effect on early neonatal death.

For interpretability, we also computed hazard ratios by exponentiating the parameters estimate. For instance, for households’ wealth, the poor ones had a hazards ratio equal to exp(-0.258) = 0.77 (95% CI = 0.648-0.922) relative to the rich ones, while holding all other variables constant. Similarly, exp(1.830) = 6.23 (95% CI = 4.522-8.589): the expected hazard is 6.23 times higher among the deceased newborn who had a severe illness at onset compared to those who had not. The latter variable is a time-varying variable that was obviously significant (**Table 3**). The assumption that newborns with severe illness or not at onset had a proportional death risks was not justified.

We repeated the same procedures for cause-specific mortality from BIBA and Infections, ie, we fitted the Cox regression model to predict the risk factors of dying from BIBA and infections, separately, before investigating the proportional hazards assumption. Second, the proportional hazards assumption was assessed by a statistical test.

**Table 5** shows the risks factors for neonatal mortality from BIBA, and also that water source (piped water or not), gender (boy vs girl), and whether the deceased had a severe illness at onset were found to violate the proportional hazards assumption, thus, were included in the group of time-varying covariates equation. The hazards ratio for mortality from BIBA was significantly lower in poor households compared to rich ones (HR = 0.59 and 95% CI = 0.369-0.950). Households with no electricity had 1.79 times higher hazards ratio compared to households with electricity (95% CI = 1.180-2.715). Newborn with severe illness at onset were 13.1 times as likely as other children to die earlier from BIBA (95% CI = 5.000-34.321). We found no evidence that the severity of illness at onset effect varies linearly with time (HR = 0.46; 95% CI = 0.187-1.114) in the model that predicted neonatal mortality by BIBA.

**Table 5:** Estimated hazards rates (with 95% confidence intervals) of neonatal death from Birth Injury/ Birth Asphyxia (BIBA), Nigeria

	Birth injury/ birth asphyxia		
**Parameter estimate**	*P*-value*	**HR (95% CI for HR) ^†^**
**Main co-variates:**			
Household in the North	-0.190	0.381	0.83 (0.541-1.265)
Household in poor wealth group	-0.525	**0.030**	0.59 (0.369-0.950)
Household in rural area	0.122	0.549	1.13 (0.758-1.685)
Household had unimproved toilet	-0.138	0.551	0.87 (0.553-1.371)
Household used no piped or improved water source	-0.225	0.349	0.80 (0.498-1.279)
Household had no electricity	0.582	**0.006**	1.79 (1.180-2.715)
Mother’s age: 30 years or more‡	0.260	0.207	1.30 (0.866-1.944)
Mother had no education	-0.139	0.548	0.87 (0.552-1.370)
Mother had high parity of 4 children or more§	-0.167	0.446	0.85 (0.551-1.300)
Mother received no ANC or no quality ANC	-0.041	0.846	0.96 (0.637-1.448)
Mother did not deliver in a health facility	-0.021	0.916	0.98 (0.665-1.442)
Mother had any labor/delivery complications‖	-0.128	0.508	0.88 (0.603-1.284)
Deceased was a baby boy	0.134	0.555	1.14 (0.732-1.788)
Deceased had a severe illness at onset	2.573	**<0.001**	13.10 (5.000-34.321)
**Time varying co-variates (TVC):**¶			
Household had no piped or improved water source	0.334	0.183	1.40 (0.854-2.284)
Deceased was a baby boy	-0.367	0.158	0.69 (0.416-1.153)
Deceased had a severe illness at onset	-0.786	0.085	0.46 (0.187-1.114)

HR – hazard ratio, CI – confidence interval

*Significant *P* values (<0.05) are in bold.

†The HR represents an increased (or decreased) risk of early neonatal death. HR>1.00 indicates increased risk of early neonatal death while HR<1.00 indicates reduced risk of early neonatal death; HR = 1 means the exposure/ characteristic/factor has no effect on early neonatal death

‡Median Maternal age at time of childbirth = 30years.

§Median number of children ever born in our sample = 4.

‖Except preterm delivery complication.

¶Variables in TVC equation interacted with ***ln(_t)***; these are variables for which the null hypothesis of zero slope on the Test of proportional hazards assumption is rejected.

There was a 7.3 (95% CI = 4.491-11.861) times increased risk of dying of infections (**Table 6**) among newborns with severe illness at onset than other newborns, and the risk of death from infections significantly and linearly decreased with time to death (***_t***) for newborns with severe illness at onset compared to other children, while maintaining other factors constant.

**Table 6:** Estimated hazards rates (with 95% confidence intervals) of neonatal death from infections, Nigeria

	Main cause of death: INFECTIONS		
**Parameter estimate**	*P*-value*	**HR (95% CI for HR)†**
**Main co-variates:**			
Household in the North	-0.004	0.977	1.00 (0.745-1.330)
Household in poor wealth group	-0.250	0.067	0.78 (0.596-1.018)
Household in rural area	0.055	0.675	1.06 (0.818-1.364)
Household had unimproved toilet	0.152	0.285	1.16 (0.881-1.539)
Household used no piped or improved water source	-0.254	0.212	0.78 (0.521-1.156)
Household had no electricity	0.097	0.440	1.10 (0.862-1.408)
Mother’s age: 30 years or more‡	-0.133	0.298	0.88 (0.682-1.124)
Mother had no education	-0.237	0.067	0.79 (0.612-1.017)
Mother had high parity of 4 children or more§	-0.062	0.631	0.94 (0.731-1.209)
Mother received no ANC or no quality ANC	-0.108	0.636	0.90 (0.574-1.403)
Mother did not deliver in a health facility	-0.051	0.828	0.95 (0.599-1.507)
Mother had any labor/delivery complications‖	-0.014	0.900	0.99 (0.789-1.231)
Deceased was a baby boy	0.022	0.835	1.02 (0.834-1.252)
Deceased had a severe illness at onset	1.988	**<0.001**	7.30 (4.491-11.861)
**Time varying co-variates (TVC):**¶			
Household used no piped or improved water source	0.132	0.230	1.14 (0.920-1.415)
Mother received no ANC or no quality ANC	0.148	0.228	1.16 (0.911-1.476)
Mother did not deliver in a health facility	-0.056	0.657	0.95 (0.740-1.210)
Deceased had a severe illness at onset	-0.732	**<0.001**	0.48 (0.360-0.644)

CI – confidence interval, HR – hazards ratio

*Significant *P*-values (<0.05) are in bold.

†HR represents an increased (or decreased) risk of early neonatal death. HR>1.00 indicates increased risk of early neonatal death while HR<1.00 indicates reduced risk of early neonatal death; HR = 1 means the exposure/ characteristic/factor has no effect on early neonatal death.

‡Median Maternal age at time of childbirth = 30years.

§Median number of children ever born in our sample = 4.

‖Except preterm delivery complication.

¶Variables in TVC equation interacted with ***ln(_t)***; these are variables for which the null hypothesis of zero slope on the Test of proportional hazards assumption is rejected.

## DISCUSSION

In this paper, we provided the estimated breakdown of deaths at different time points in the neonatal period in Nigeria from 2009 to 2013. The most significant results are discussed below.

Our study confirmed that there is a high proportion of neonatal deaths in the first 3 days of life, and in particular, the first day: approximately 26% of all neonatal deaths (37.0% in the South, vs 21.4% in the North) occurred during the first day of life, consistent with previous reports that estimated that neonatal deaths on the first day ranged from 25%-45% [2,3]; and 73.9% during the first week of life [6,21,22]. Reducing early neonatal mortality would not only require management and care of potential complications, such as preeclampsia, maternal infections, prolonged labor/obstructed delivery and other delivery complications, but also the implementation of basic neonatal resuscitation intervention to initiate spontaneous respiration. Given the fact that the country has shown a slow rate of decline in under-five mortality over the past 15 years [1], these findings emphasize an urgent need for policies and programs that specifically target neonate s during the first week of life. Interventions such as quality antenatal care and institutional delivery by a skilled attendant are likely also still needed [22].

Another finding was that a quarter (25.9%) and 80.3% of neonatal deaths in the first two days of life are secondary to infections, likely including sepsis, meningitis, pneumonia and BIBA. Needless to say, the high proportion of deaths due to BIBA in the two days after birth echoes the interventions the immediate postnatal period as stated above. It is also well known that most of these infection-related deaths are caused by the pathogens vertically transmitted from the mother. This emphasizes the importance of screening of pregnant women and treatment of asymptomatic bacteriuria (ASB) and urinary and reproductive tract infections [23].

Recently there has been a growing demand for neonatal mortality data to be disaggregated by gender, geographic location, and socioeconomic status, to enable programs improve resource allocation and monitoring [24]. Thus, we investigated factors that increased the risk of early death among the deceased newborns. Just a few variables passed the significance test.

Among the deceased newborns, those in poor households died at older ages from all causes and BIBA compared to those in wealthier households. We suspect that, because of underreporting, actual Nigerian neonatal mortality rates may be higher among poor households than reported.

In addition, this study showed that poor households had lower hazards ratio for mortality from BIBA, while households with no electricity had 1.79 times higher hazards ratio compared to households with electricity. These results seemed at first to be counterintuitive as one would expect poor households to also lack or not to be able to afford other basic commodities such as electricity. But for years, the country has been suffering from a chronic and seemingly intractable power supply problem. It is estimated that about 90 million Nigerians, or 50% of the country population, lack electricity [9]. Therefore, in places where energy supply is weak, use of electricity would not make a difference between poor and wealthier households. In any case, the absence of electricity in the household as shown in the study, was significantly and positively linked to early neonatal death from BIBA. One explanation could be that availability of electricity may help to create better environmental conditions in the house for the newborn [21]. It not only helps in hygienic preparation of food, but also encourages the use of television and radio as instruments of information and education for health seeking behavior change. Lastly, our study supported the claim that, in case of non-proportional hazards, extended Cox regression models that allow time-dependent variables could be more appropriate than the simple Cox regression model [19].

In the coming years, there is the need to accelerate the impressive progress being made in reducing preventable child deaths, including the burden of nearly 3 million neonatal deaths that are most frequent within the first week of life. Further research is needed to better understand the social, cultural, and logistical factors that are driving high mortality in the early days following delivery.

Nonetheless, our study has limitations inherent to its cross-sectional design and some have been discussed in prior publications [6,10] and include the long recall period that had been ameliorated by the quality of the trainings and the trainees. Additionally, recall errors arising from dates of birth and death given by women interviewed in the survey were minimized by restricting our analyses to births within the 5-year period preceding the survey. The platform NDHS interviewed only surviving women to collect the full birth history data, which may have led to under-reporting of the number of newborn deaths because of the association of neonatal death with maternal death [23]. Even though several studies have found VA to be a viable method to determine the most important causes of neonatal death in developing countries [13,14,16], it is not the gold standard. We included several risk factors in the multivariate analyses, yet a very few of them were significant. This is not to say that other non-significant risk factors were not associated with all-cause or other cause-specific mortality; their lack of significance might be due to confounding, eg, interrelationships among the risk factors considered. Lastly, an analysis including representative data for each of the six geopolitical zones in Nigeria might have produced study results with implications for improvement of local maternal and neonatal health programs.

## CONCLUSIONS

This paper aimed to gain insights from the rich VASA data set on the timing, causes and determinants of neonatal mortality in Nigeria, including some maternal conditions, that would not otherwise be available. Methodologically, adapting survival analysis to neonatal time to death is an important and dynamic research area. The Cox regression models that can be used in the presence of nonproportional hazards were considered in this study. The analysis yielded some results that are not entirely new, such as that the majority of neonatal deaths occur in the first days of life, thus suggesting the need to improve the quality of care at birth and in the immediate postnatal period. The analyses may also require confirmation in real-world cohorts with detailed, time constant or varying social, cultural, and logistical factors that are driving high mortality in the early days following delivery.
